# American Academy of Pediatrics 2014 Bronchiolitis Guidelines: Bonfire of the Evidence

**DOI:** 10.5811/westjem.2015.1.24930

**Published:** 2015-01-12

**Authors:** Paul Walsh, Stephen J. Rothenberg

**Affiliations:** * University of California, Davis, Department of Emergency Medicine, Davis, California; † Sutter Medical Centers of Sacramento, Pediatric Emergency Medicine, Sacramento, California; ‡ Instituto Nacional de Salud Pública, Centro de Investigación en Salud Poblacional, Cuernavaca, Morelos, Mexico

## A BONFIRE OF THE EVIDENCE

The American Academy of Pediatrics (AAP) 2014 Bronchiolitis guidelines (the guidelines) were recently published in the official journal of the AAP, Pediatrics.^1^ The committee that wrote the guidelines anticipates that these will form the basis of bronchiolitis treatment throughout the house of medicine, not just in pediatricians’ offices. Emergency physicians may well encounter pressure to follow these guidelines from their pediatric colleagues who, not unreasonably, rely on guidelines from their professional organization.

However, two key recommendations in these guidelines could substantially change pediatric emergency medicine practice. These recommendations are (1) to not use even a trial of bronchodilators and (2) to regard oxygen saturations of 90% rather than 92%–94% as the degree of hypoxia at which oxygen should be administered.^1^ Neither of these recommendations is sufficiently justified by the evidence and both are potentially harmful. We deal first with the new guideline to not use bronchodilators.

The committee bases its recommendation to not attempt even a trial of bronchodilators on the following:

1. The committee’s interpretation of a meta-analysis that reported a decrease in hospital admissions when epinephrine rather than placebo was given in the emergency department (ED).^1^
2. A meta-analysis contained in a Cochrane review, which did not show decreased hospital admissions from the ED when albuterol rather than placebo was given.^2^
3. Albuterol non-responders cannot be distinguished from responders, and clinicians’ ability to observe a clinically relevant response to bronchodilators is limited.^1^
4. Albuterol’s risks and expense outweigh its benefits.^1^

We deal with each of these in turn. Bronchiolitis causes lower airway obstruction through a combination of bronchiolar obstruction with inflammatory cells, cellular debris, increased mucus secretion, and varying degrees of bronchospasm. This combination has prompted treatment with nebulized epinephrine, which can decrease mucosal edema and has bronchodilator properties, and albuterol, which is best known for its bronchodilator properties (Footnote[a]).^3^–^5^

A meta-analysis found a decrease in hospital admissions from the ED risk ratio 0.67 (95% CI [0.50–0.89]) favoring epinephrine over placebo.^6^ This analysis was heavily influenced by Plint et al., which recruited 800 patients divided into four groups comparing combinations of epinephrine, saline, dexamethasone, and placebo and found early benefits but little difference at one week between nebulized epinephrine and normal saline.^7^ Both this meta-analysis and Plint et al. were published by the same group, and as reported the meta-analysis would have justified further funding for additional studies.^6^

However, this meta-analysis excluded another large randomized controlled trial (RCT) comparing albuterol and epinephrine.^6^ Walsh et al. randomized 703 patients in two groups comparing nebulized albuterol and epinephrine.^83^ This study found a relative increase in ED discharge of 18% when albuterol rather than epinephrine was used (aRR 1.18 for successful ED discharge without admission at three days follow up).^8^ This is equivalent to a risk ratio of 0.86 (95% CI [0.76–0.98]) for decreased admission. Since an adequately powered large RCT had already demonstrated decreased admissions from the ED when albuterol rather than epinephrine is used, neither the meta-analysis nor another RCT were needed. Contrary to the committee’s assertions, the data show progressively decreasing admissions from the ED when nebulized normal saline, epinephrine, or albuterol are used in treatment.

The second rationale relied on by the committee to recommend against the use of albuterol is a meta-analysis contained in a Cochrane review performed by Gadomski et al. This meta-analysis reported an OR 0.77, (95% CI [0.44–1.33]) for hospital admission from the ED.^2^ This null result was interpreted by the guideline authors as ‘clearly negative.’^1^,^9^ Such an interpretation is unfortunate: the statistical power of this analysis to detect a relative decrease of 20% in admission was 18% (n=404 with the reported sample characteristics, α=0.05). A null result in an inadequately powered study is no basis for concluding a drug has no effect.

The committee attached particular weight to placebo-controlled studies, which it regards as ‘the highest form of evidence,’ and therefore excluded studies that compared bronchodilators from their deliberations. However, when placebo is not the standard of care then placebo is not necessarily the best or even correct comparator.^10^ To demonstrate this effect we have recreated the meta-analysis relied on by the committee, this time including the largest excluded study which did show a benefit to using albuterol in the ED. (We conservatively assumed epinephrine to be no more effective than placebo, and used relative risk rather than odds ratios because hospital admission is not rare and risk is easier to interpret.) The result (Figure) shows that albuterol treatment of bronchiolitis in the ED leads to decreased admissions and how little underpowered studies contribute to our knowledge.

We disagree with both components of the committee’s third rationale for not using bronchodilators. First, the assertion that albuterol non-responders cannot be distinguished from responders is inaccurate. A therapeutic trial distinguishes them handily. Second, the committee’s assertion that clinicians are unable to adequately observe clinically relevant responses to bronchodilators ignores the reality that emergency physicians are highly experienced in the management of bronchospasm and the use of bronchodilators. The recommendation that albuterol be withheld from everyone with bronchiolitis because it may prevent admission in only a minority,^1^,^9^ denies clinicians the common sense practice of the therapeutic trial. If the child responds to albuterol it can be continued; if not, it can be discontinued.

We also disagree with the committee’s fourth rationale for recommending against the use of bronchodilators, namely their assessment of the dangers and expense of albuterol.^1^ Albuterol in reasonable doses has a long record of safety in infants and children; we even allow primary school children to carry and self-administer it. And premixed albuterol ampoules retail for 36 cents/dose at a large multipurpose national retailer. The 18% relative reduction in hospital admissions from the ED that can be obtained using albuterol is surely also an important part of any cost-benefit calculation.^8^

Other studies, including a Cochrane review meta-analysis cited by the committee as evidence against using albuterol, in fact demonstrate that albuterol in the ED significantly improves clinical scores.^2^ Clinical scores reflect respiratory distress, which certainly seems worth relieving. Not all cases of this short-term relief of respiratory distress will translate into decreased hospital admissions. But some will. This evidence has been ignored in formulating the current guidelines.^1^

The second recommendation which emergency physicians might best ignore is that clinicians may withhold supplemental oxygen if the oxygen saturation is ≥90% rather than the 92% used elsewhere. The committee writing the guidelines base this recommendation on ‘low level evidence and reasoning from first principles.’^1^ The committee’s rationale is that:

1. Oxygen saturations of 90% are not materially different from oxygen saturations of 92%.
2. The Collaborative Home Infant Monitoring Evaluation (CHIME) study found that oxygen desaturations commonly occur in the sleep of normal infants without ill effect.^11^

This recommendation appears to discount the fact that the normal range of oxygen saturation for this age group at sea level is 97%–100%.^12^ It also ignores evidence that a pulse oximeter reading of 90% tends to overestimate the actual oxygen saturation in children (mean bias 4.2% between 86% and 90% and 1.8% between 91%–95%).^13^

There is uncertainty as to what level and duration of hypoxia is harmful in infants in general and bronchiolitis in particular. Increasing altitude increases the odds of being at risk for neurodevelopmental problems (100-meter increase in altitude: OR= 1.02; 95% CI [1.001–1.037] after adjustment for other factors).^14^ A detailed systematic review of the literature on hypoxia in children found causal evidence for adverse effects of chronic and intermittent (as can occur in snoring/sleep disorders) hypoxia in children. These adverse effects included decreased intelligence quotient (IQ), neurocognitive functioning, and increases in behavioral disorders and attention deficit hyperactivity disorder symptoms when oxygen saturation even intermittently ranges from 90%–94%. These associations are insufficient to prove causality, but these same adverse effects were also found for hypoxia related to asthma and respiratory instability in infants.^15^

The CHIME study found transient oxygen desaturation during sleep is not uncommon in infants and appears to have little adverse effect.^7^ However these transient oxygen desaturations were short: ≤6 seconds duration. When hypoxia occurs in bronchiolitis it can be expected to be present for hours or days, not seconds. The CHIME study is simply not pertinent.

Knowing that even relatively mild hypoxia (90%–94%) may have long-term sequelae in infants, and knowing that the duration of hypoxia of acute bronchiolitis is likely to be to be prolonged, it is difficult to justify withholding oxygen. Sensible oxygen administration that avoids hyperoxia is not risky. Whether one should choose an oxygen saturation treatment threshold of 92% or 94% in previously healthy infants is worthy of discussion; 90% is probably too low. Studies of neurocognitive function in at least some infants with treated and untreated hypoxia from bronchiolitis have not been carried out nor are they likely to be. Waiting for such studies as the committee appears to be doing strikes us as unwise. However, we can anticipate that in infants, many of whom will be less than four months old and may still have fetal hemoglobin, the low Pa02 associated with an Sa02 of 90% will fall yet further after discharge.

These recommendations within the guidelines seem to be premised on an underlying belief that because bronchiolitis is a short-lived generally non-fatal disease, treatment cannot offer long-term benefit, and that most treatment should therefore be avoided. Emergency physicians’ *raison d’être* however is to treat acute conditions; relieving acute respiratory distress and hypoxia using interventions as simple as albuterol and oxygen is not only good emergency medicine practice; it is in fact supported by the available evidence.

## Figures and Tables

**Figure:**
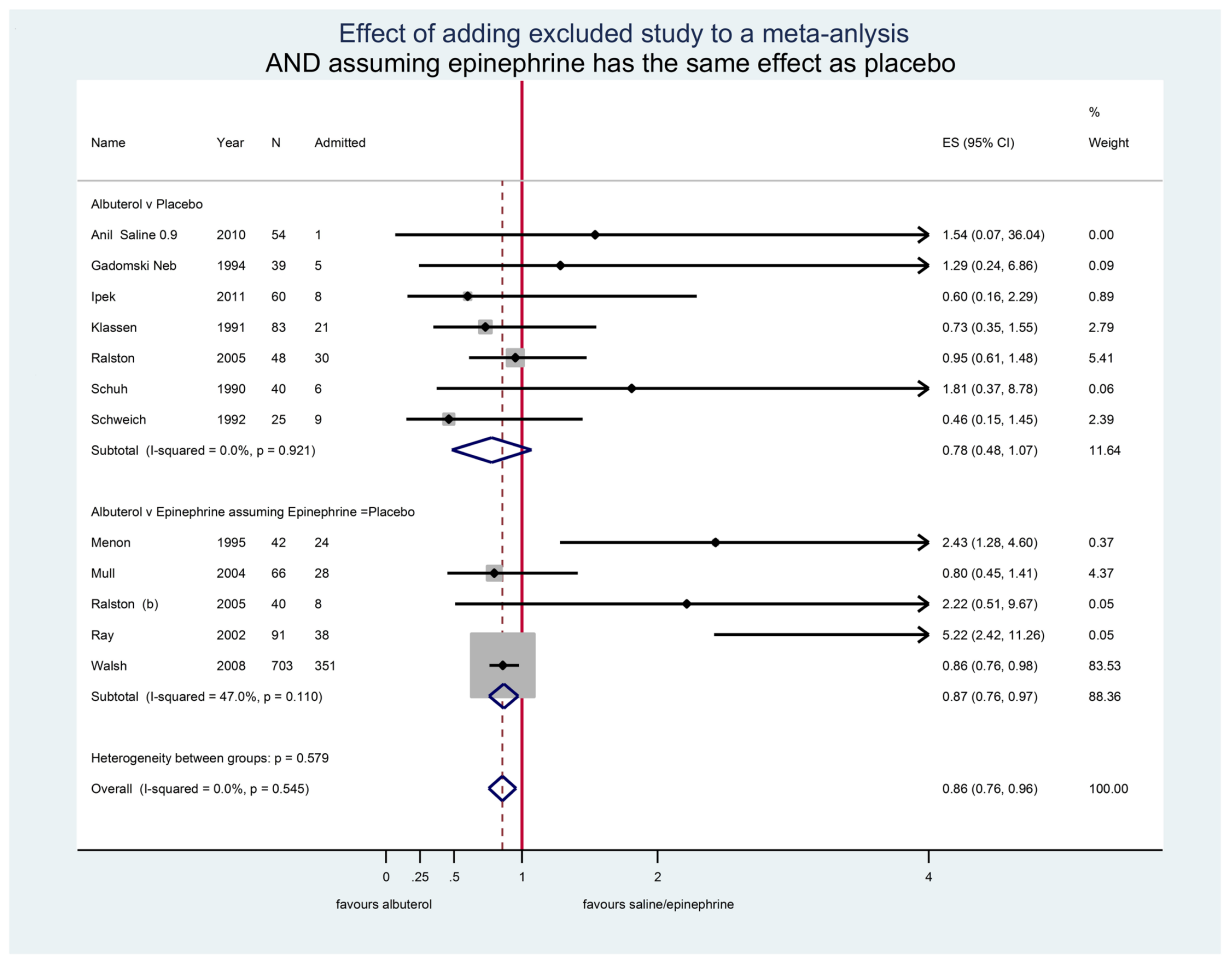
The purpose of this Forest plot is to show the effect of excluding a single large randomized controlled trial and how little information is actually contained in smaller ones. The top analysis reproduces the meta-analysis of Gadomski et al. The boxes reflect study weight which is a function of study size and the number of events (admissions). In both comparisons studies showing a benefit to albuterol have narrower confidence intervals reflecting the greater precision of these studies. *ES*; effect size as relative risk of discharge ^a^ Steroids do not generally decrease hospital admission from the emergency department, although steroids may have a role in recurrent episodes if there is a family history of asthma. Factors other than simple bronchodilation may also play a role in albuterol’s effect. ^b^ Includes two (albuterol and 0.9% normal saline and epinephrine and 0.9% normal saline) of the five arms of the original study without penalizing any arm. ^c^ Includes two of the three arms of the study, again without penalizing the epinephrine/placebo arm.
